# ROS1-mediated decrease in DNA methylation and increase in expression of defense genes and stress response genes in *Arabidopsis thaliana* due to abiotic stresses

**DOI:** 10.1186/s12870-022-03473-4

**Published:** 2022-03-07

**Authors:** Liping Yang, Chenjing Lang, Yanju Wu, Dawei Meng, Tianbo Yang, Danqi Li, Taicheng Jin, Xiaofu Zhou

**Affiliations:** 1 The School of Life Sciences, Jilin Normal University, Siping, China; 2 The School of Life Sciences, Northwest A&F University, Xianyang, Shanxi China; 3 The School of Life Sciences, Jilin University, Changchun, China

**Keywords:** DNA methylation, Salicylic acid, ROS1-mediated DNA demethylation, Abiotic stresses

## Abstract

**Background:**

Small interfering RNAs (siRNAs) target homologous genomic DNA sequences for cytosine methylation, known as RNA-directed DNA methylation (RdDM), plays an important role in transposon control and regulation of gene expression in plants. Repressor of silencing 1 (ROS1) can negatively regulate the RdDM pathway.

**Results:**

In this paper, we investigated the molecular mechanisms by which an upstream regulator ACD6 in the salicylic acid (SA) defense pathway, an ABA pathway-related gene *ACO3*, and *GSTF14*, an endogenous gene of the glutathione S-transferase superfamily, were induced by various abiotic stresses. The results demonstrated that abiotic stresses, including water deficit, cold, and salt stresses, induced demethylation of the repeats in the promoters of *ACD6*, *ACO3*, and *GSTF14* and transcriptionally activated their expression. Furthermore, our results revealed that ROS1-mediated DNA demethylation plays an important role in the process of transcriptional activation of *ACD6* and *GSTF14* when *Arabidopsis* plants are subjected to cold stress.

**Conclusions:**

This study revealed that ROS1 plays an important role in the molecular mechanisms associated with genes involved in defense pathways in response to abiotic stresses.

**Supplementary Information:**

The online version contains supplementary material available at 10.1186/s12870-022-03473-4.

## Background

DNA methylation is one of the most common forms of covalent DNA modification in the genomes of eukaryotes and plays an important role in the growth and development of plants and in responses to various abiotic stresses. RNA silencing is a conserved pathway that results in the blockage of gene expression in both the cytoplasm and nucleus of eukaryotic organisms [1]. In plants, small interfering RNAs (siRNAs) target homologous sequences for DNA methylation, a process known as RNA-directed DNA methylation (RdDM); this process plays an important role in regulating gene expression, controlling the activity of transposable elements, and defending against foreign DNAs, such as DNA viruses [2–4]. These siRNAs are synthesized by RNA polymerase IV (Pol IV), RNA-dependent RNA polymerase (RDR2), and Dicer-like 3 (DCL3) [5]. Argonaute protein 4 (AGO4) and the DNA methyltransferases DRM1/2, MET1, and CMT3 perform de novo methylation and maintain methylation of the target DNA [6]. DNA methylation can be reversed by DNA glycosylases/lyases in *Arabidopsis* plants, and this process is known as active demethylation [7]. Repressor of silencing 1 (ROS1) can negatively regulate the RdDM pathway [8, 9]. ROS1-mediated DNA demethylation helps determine genomic DNA methylation patterns and protects active genes from being silenced [10].

Abiotic stresses mainly include drought, cold, and salt stresses, which severely threaten plant growth and crop yields [11, 12]. Abiotic stresses can induce the accumulation of endogenous abscisic acid (ABA), triggering ABA signal transduction to cope with adverse environmental factors [13–15]. When plants are under cold stress, ABA can regulate the expression of cold resistance genes in plants in response to stress [16–18]. Abiotic stress also affects dynamic changes in DNA methylation in plants. Changes in methylation levels and patterns regulate the expression of stress-responsive genes, thereby improving the resistance of plants to stress [19]. In *Arabidopsis*, the *ros1* mutant is hypersensitive to ABA, and ROS1 participates in the ABA response by regulating the expression of NICOTINAMIDASE 3 (NIC3) [20]. Soybean has been found to show abnormal expression of approximately 49 transcription factors under salt stress, and the expression profiles of the MYB, b-ZIP, and AP2/DREB transcription factor families are reportedly significantly correlated with the DNA methylation of their gene sequences [21]. Abiotic stress can regulate the expression of stress-responsive genes by inducing dynamic changes in DNA methylation, thereby improving the adaptability of plants to the environment.

Salicylic acid (SA) is an important signaling molecule in plant defense responses and can induce the expression of defense genes and the development of systemic resistance [22]. At least three types of SA regulators have been described [23]: type I regulators, including enzymes involved in SA biosynthesis, e.g., SA INDUCTION-DEFICIENT 2 (SID2) [24], type II regulators such as accelerated cell death 6 (ACD6), which are upstream from SA [25–27], and type III regulators, which transduce signals downstream from SA, e.g., NONEXPRESSOR OF PR GENES 1 (NPR1) [28]. A gain-of-function mutant of *ACD6* (*acd6–1*) has been reported to increase the expression of defense genes in the SA pathway [29]. Plants respond to pathogens via the SA, jasmonic acid (JA), and ethylene (ET) pathways [2]. The role of SA in plant tolerance to various biotic stresses has been intensively studied [30]. SA also plays an important role in modulating plant responses to many abiotic stresses, including salt, drought, and chilling [31]. For example, salinity induces increases in endogenous SA levels and the activity of the SA biosynthesis enzyme in rice seedlings [32]. Our previous study revealed the molecular mechanisms underlying the induction of defense genes in the SA pathway by biotic stresses in *Arabidopsis* plants [4, 33]. However, the regulatory mechanisms of genes involved in defense pathways in response to abiotic stresses remain unclear.

In this study, we determined the molecular mechanisms underlying the functioning of the upstream regulator ACD6 of the SA pathway, the endogenous gene *GSTF14* in the glutathione S-transferase (GST) superfamily, and aconitate hydratase 3 (ACO3) in response to abiotic stresses. The results showed that the expression levels of defense genes (*ACD6*, *NPR1*, and *PR5*) in the SA pathway, *ACO3*, and *GSTF14* significantly increased after exposure to water deficit, cold, and salt stresses. Sequencing results confirmed that abiotic stresses induced demethylation of the repeats in the promoters of *ACD6*, *ACO3*, and *GSTF14* and transcriptionally activated their expression. Further experiments revealed that ROS1-mediated DNA demethylation plays an important role in the mechanisms of these defense genes in response to abiotic stresses.

## Results

### Induction of SA pathway-related defense genes by abiotic stresses

**Fig. 1:**
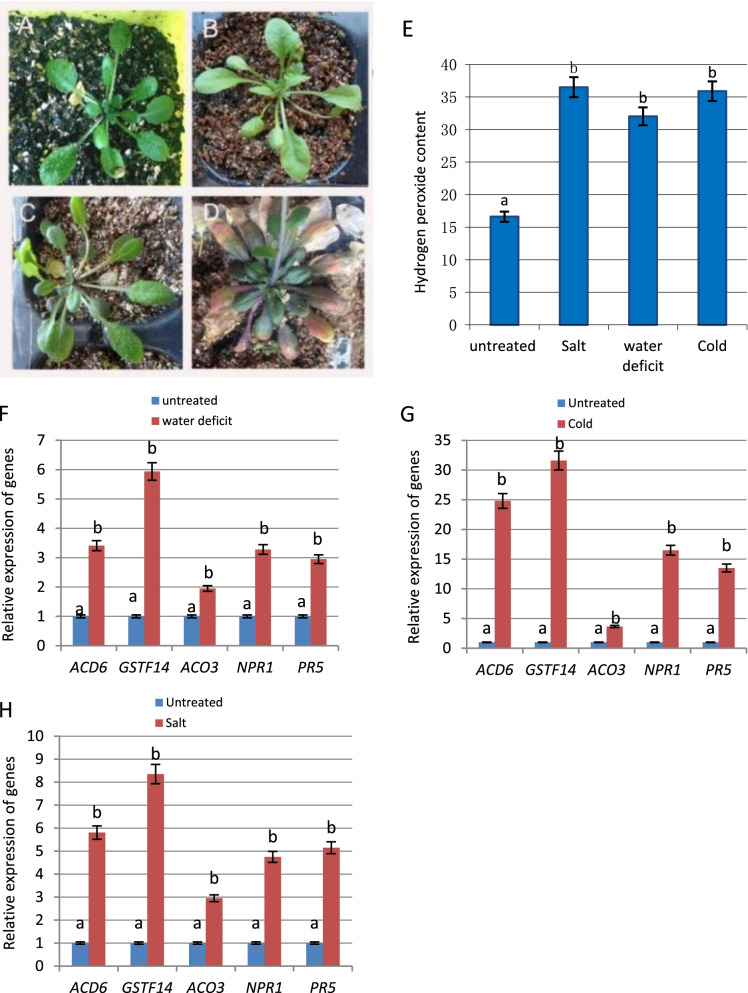
Detection and analyses of the expression of defense genes and stress response genes in *Arabidopsis* plants treated with abiotic stresses. **A** The untreated *Arabidopsis* Col-0 plants. **B**, **C** The leaves of *Arabidopsis* plants treated with water deficit stress turned slightly yellow and shrunk during days 5–7. **D** Anthocyanin accumulation in the leaves of *Arabidopsis* plants treated with water deficit stress clearly increased, and the leaves turned severely yellow and withered on day 14. **E** The measurements of hydrogen peroxide content were performed by spectrophotometry, the results showed that hydrogen peroxide content increased in Col-0 plants after cold stress (4 °C) for 24 h, water deficit for 7 days or salt stress (150 mM) for 3 days. **F** The transcript levels of related and defense genes in *Arabidopsis* plants treated with water deficit stress were analyzed by qPCR. **G** The transcript levels of defense genes, *GSTF14*, and *ACO3* in *Arabidopsis* plants treated with cold stress were analyzed by qPCR; untreated Col-0 plants served as controls. **H** The transcript levels of defense genes, *GSTF14,* and *ACO3* in *Arabidopsis* plants treated with salt stress were analyzed by qPCR; untreated Col-0 plants served as controls. Means identified by different letters are significantly different from each other. Error bars represent SEs from three biological replicates. One-way ANOVA followed by Tukey’s Multiple comparison Test was used for statistical analysis. (*P* < 0.05)

Our previous studies verified an upstream regulator (ACD6) in the SA pathway, and *GSTF14*, an endogenous gene of the glutathione S-transferase superfamily that is implicated in numerous stress responses, which revealed the molecular mechanism underlying the induction of defense gene expression in the SA pathway by biotic stresses [4, 33]. To investigate whether abiotic stress can induce the expression of *ACD6*, *GSTF14*, and an ABA pathway-related gene (*ACO3*), the wild-type Columbia (Col-0) line of *Arabidopsis thaliana* was selected for water deficit treatment, cold stress treatment, and salt stress treatment. On days 5–7, the leaves of Col-0 plants treated with water deficit stress turned slightly yellow and shrunk (Fig. 1B, C) compared to those of untreated Col-0 plants (Fig. 1A). On day 14, anthocyanin accumulation in the leaves of Col-0 plants treated with water deficit stress clearly increased, and the leaves turned severely yellow and withered (Fig. 1D). No significant phenotypic changes were observed in plants treated with cold stress (4 °C) for 24 h or salt stress (150 mM) for 3 days. Abiotic stresses make a large amount of Reactive Oxygen Species (ROS) accumulate in plant cells. ROS content can be served as a kind of stress makers, including hydrogen peroxide content and superoxide anino. To confirm that Col-0 plants treated under different conditions were indeed stressed, we performed the measurements of hydrogen peroxide content by spectrophotometry. The results showed that hydrogen peroxide content significantly increased in Col-0 plants after cold stress (4 °C) for 24 h, water deficit for 7 days or salt stress (150 mM) for 3 days (Fig. 1E) and confirmed the treated plants were under the specific stress conditions.

We extracted total RNA from wild-type *Arabidopsis* Col-0 plants on the 7th day of water deficit treatment for comparative analysis of gene expression. Quantitative reverse transcription-polymerase chain reaction (RT-qPCR) analysis confirmed that *ACD6*, *GSTF14*, and *ACO3* were significantly upregulated after water deficit treatment, and the upregulation of *GSTF14* expression was more pronounced (Fig. 1F). Since ACD6 is an upstream regulator of the SA pathway, the increase in *ACD6* expression may upregulate the expression of the defense genes *NPR1* and *PR5* (Fig. 1F). To further investigate whether cold or salt stress can also induce the expression of defense genes in the SA pathway, we analyzed the expression of related genes in untreated Col-0 plants and Col-0 plants treated under different conditions. The results showed that compared with controls, Col-0 plants treated with cold or salt stress had significantly higher expression levels of defense genes *ACD6*, *NPR1*, *PR5*, and stress response genes *GSTF14* and *ACO3* and confirmed that cold stress and salt stress activated *ACD6* expression, which was significantly increased after 24 h of cold stress treatment (Fig. 1G, H).

### Direct correlation between the increased expression of defense and stress resistance genes and the reduction in promoter DNA methylation

**Fig. 2:**
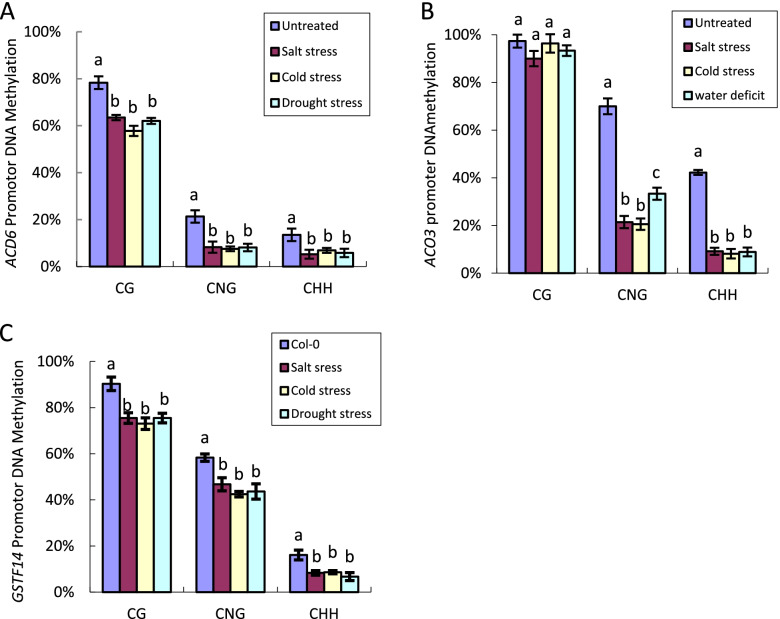
Analyses of DNA methylation in promoters in plants treated with different stresses. **A** Percentage of DNA methylation in the repeat regions of the ACD6 promoter in plants treated with different stresses and untreated Col-0 plants. **B** Percentage of DNA methylation in the repeat regions of the ACO3 promoter in plants treated with different stresses and untreated Col-0 plants. **C** Percentage of DNA methylation in the repeat regions of the GSTF14 promoter in Col-0 plants treated with different stresses and untreated Col-0 plants. Fifteen individual clones of each genotype were used for sequencing. Means identified by different letters are significantly different from each other. Error bars represent SEs from three biological replicates. One-way ANOVA followed by Tukey’s Multiple comparison Test was used for statistical analysis (*P* < 0.05)

To determine whether upregulation of these genes is correlated with a decrease in methylation at these genes’ promoters, the DNA methylation data of these genes were first searched at http://epigenomics.mcdb.ucla.edu/BS-Seq/ [33, 34]. Extensive methylation was found in the promoter sequences corresponding to the promoter regions of *ACD6* and *ACO3*, and strong methylation corresponding to the promoter region of *GSTF14* was found through a methylation pattern search. Col-0 plants were treated with abiotic stresses, and cytosine methylation in the gene promoter regions were analyzed with a bisulfite sequencing method. The data revealed that the DNA methylation levels in the region of the *ACD6* promoter was reduced by 16.27% (a change from 78.30 to 62.03%) in CG sites, by 13.56% (a change from 21.67 to 8.11%) in CNG sites, and by 7.71% (a change from 13.51 to 5.80%) in CHH sites in Col-0 plants treated with water deficit stress. The DNA methylation levels in the region of the *ACD6* promoter was reduced by 20.55% (a change from 78.32 to 57.77%) in CG sites, by 14.11% (a change from 21.67 to 7.56%) in CNG sites, and by 8.15% (a change from 13.51 to 5.36%) in CHH sites in Col-0 plants treated with cold stress. The DNA methylation levels in the region of the *ACD6* promoter was reduced by 14.86% (a change from 78.32 to 63.46%) in CG sites, by 13.41% (a change from 21.67 to 8.26%) in CNG sites, and by 8.26% (a change from 13.51 to 5.25%) in CHH sites in Col-0 plants treated with salt stress (Fig. 2A).

The data revealed that CG methylation of the repeats in the *ACO3* promoter was not significantly altered, while DNA methylation in the *ACO3* promoter was reduced by 32.56% (a change from 65.89 to 33.33%) in CNG sites and by 33.33% (change from 42.22 to 8.89%) in CHH sites in Col-0 plants treated with water deficit stress. The DNA methylation levels in the *ACO3* promoter was significantly reduced by 32.56% (a change from 65.89 to 20%) in CNG sites and by 33.33% (a change from 42.22 to 8.16%) in CHH sites in Col-0 plants treated with cold stress. DNA methylation in the *ACO3* promoter was significantly reduced by 44.46% (a change from 65.89 to 21.43%) in CNG sites and by 33.03% (a change from 42.22 to 9.19%) in CHH sites in Col-0 plants treated with salt stress (Fig. 2B). DNA methylation in the *GSTF14* promoter was also analyzed using bisulfite sequencing. DNA methylation of the repeats in the *GSTF14* promoter was reduced by 14.81% (a change from 90.30 to 75.49%) in CG sites, by 15.43% (a change from 64.04 to 48.61%) in CNG sites, and by 12.06% (a change from 20.78 to 8.72%) in CHH sites in Col-0 plants treated with water deficit stress. DNA methylation of the repeats in the *GSTF14* promoter was reduced by 17.27% (a change from 90.30 to 73.03%) in CG sites, by 12.58% (a change from 64.04 to 51.46%) in CNG sites, and by 12.58% (a change from 20.78 to 9.63%) in CHH sites in Col-0 plants treated with cold stress. DNA methylation in the GSTF14 promoter was reduced by 14.80% (a change from 90.30 to 75.50%) in CG sites, by 11.29% (a change from 64.04 to 52.75%) in CNG sites, and by 12.43% (a change from 20.78 to 8.35%) in CHH sites in Col-0 plants treated with salt stress (Fig. 2C).

### Role of ROS1 in regulation of the SA pathway in response to abiotic stresses

**Fig. 3:**
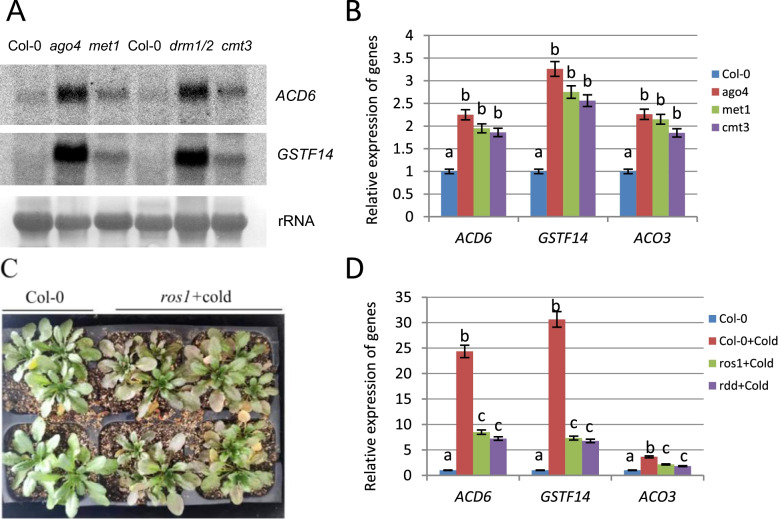
Analyses of the expression levels of related genes. **A** Analyses of the expression levels of *ACD6* and *GSTF14* in the mutants *ago4*, *met1*, *drm1/2*, and *cmt3* by northern blotting; wild-type Col-0 ecotypes served as background controls for the mutant genotypes. **B** Analyses of the expression levels of *ACD6*, *ACO3*, and *GSTF14* by RT-qPCR in DNA methylation mutant plants *ago4*, *met1*, and *cmt3* with wild-type plants as a background control for the mutant genotypes. **C** The cold stress-treated ros1 mutants exhibited deformed leaves and increased anthocyanin accumulation on the 7th day compared with the cold stress-treated Col-0 plants. **D** The related genes were analyzed in untreated Col-0 plants, Col-0 plants treated with cold stress, and *ros1* and *ros1 dml2 dml3* (*rdd*) mutant plants treated with cold stress by RT-qPCR. Means identified by different letters are significantly different from each other. Error bars represent SEs from three biological replicates. One-way ANOVA followed by Tukey’s Multiple comparison Test was used for statistical analysis (*P* < 0.05)

To further study the molecular mechanisms underlying the functioning of defense genes of the SA pathway in response to abiotic stresses, we used RNA gel blotting to analyze the expression of related genes in plants mutated at key functional elements of the RdDM pathway. The results showed that *ACD6* and *GSTF14* expression clearly increased in the mutant *ago4* and DNA methyltransferase mutants *met1*, *drm1/2*, and *cmt3* with Col-0 ecotypes as controls (Fig. 3A). RT-qPCR results further confirmed that *ACD6*, *GSTF14*, and *ACO3* were upregulated in the *ago4* mutant (Fig. 3B), indicating that RdDM has an important role in maintaining the low transcription levels of *ACD6*, *GSTF14*, and *ACO3* in wild-type plants; however, these mutants showed increased transcript levels for those genes.

ROS1 can negatively regulate the RdDM pathway [8, 9]. To determine whether ROS1 plays a role in the responses of these genes to abiotic stress, we applied cold stress treatment to loss-of-function *Arabidopsis ros1* mutants and *Arabidopsis* Col-0 plants. Under normal growth conditions, the *ros1* mutants showed no obvious developmental defects compared to Col-0 plants. Compared with the cold stress-treated Col-0 plants, the cold stress-treated *ros1* mutants appeared to exhibit more severely deformed leaves and increased anthocyanin accumulation on the 7th day (Fig. 3C), indicating that the *ros1* mutants exhibited increased susceptibility to cold stress. We further compared the expression of the *ACD6* gene between cold stress-treated *ros1* mutants (ros1 + cold) and cold stress-treated Col-0 (Col-0 + cold) plants. The results showed that *ACD6* expression in the cold stress-treated Col-0 plants significantly increased, compared with that in untreated Col-0 plants. However, the increase in *ACD6* expression in the cold stress-treated *ros1* mutants and loss-of-function *ros1dml2dml3* (*rdd*) mutants was partially inhibited compared with that in the cold stress-treated Col-0 plants (Fig. 3D). ROS1 plays an important role in the activation of defense genes in response to abiotic stress, which was confirmed by the expression levels of *GSTF14* and *ACO3*. When cold stress-treated Col-0 plants were used as the control, the increase in *GSTF14* and *ACO3* expression was partially inhibited in the cold stress-treated *ros1* mutants (Fig. 3D).

### ROS1-mediated decrease in DNA methylation of genes under abiotic stresses

**Fig. 4:**
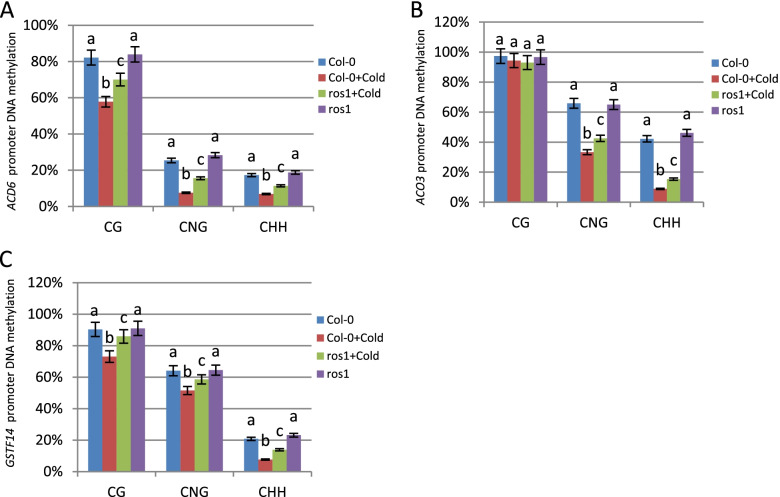
Analyses of DNA methylation of related genes. **A** Analyses of DNA methylation in the repeat regions of the *ACD6* promoter in Col-0 plants, *ros1* mutants, and Col-0 plants and *ros1* mutants treated with cold stress. **B** Analyses of DNA methylation in the repeat regions of the *ACO3* promoter in Col-0 plants, *ros1* mutants, and Col-0 plants and *ros1* mutants treated with cold stress. **C** Analyses of DNA methylation in the repeat regions of the *GSTF14* promoter in Col-0 plants, *ros1* mutants, and Col-0 plants and *ros1* mutants treated with cold stress. Fifteen individual clones of each genotype were used for sequencing. Error bars represent SEs from three biological replicates. Means identified by different letters are significantly different from each other. One-way ANOVA followed by Tukey’s Multiple comparison Test was used for statistical analysis (*P* < 0.05)

Sequencing analysis demonstrated that the DNA methylation levels of the repeats in the *ACD6* promoter in cold stress-treated Col-0 plants were significantly reduced compared with untreated Col-0 plants, including the CG, CNG, and CHH sites, while the decrease in DNA methylation levels of the repeats in the *ACD6* promoter in cold stress-treated *ros1* mutants was partially inhibited (Fig. 4A). When Col-0 plants were used as the control, the DNA methylation levels of the repeats in the *ACD6* promoter in *ros1* mutants were not significantly altered (Fig. 4A).

The results demonstrated that DNA methylation at CNG and CHH sites in the *ACO3* promoter in cold stress-treated Col-0 plants was significantly decreased compared with untreated Col-0 plants, while the decrease in DNA methylation at CNG and CHH sites in the *ACO3* promoter in cold stress-treated *ros1* mutants was partially inhibited (Fig. 4B). When Col-0 plants were used as the control, the DNA methylation levels of the repeats in the *ACO3* promotor in *ros1* mutants were not significantly altered (Fig. 4B).

The results further confirmed that the DNA methylation levels in the *GSTF14* promoter in cold stress-treated Col-0 plants were significantly decreased compared with untreated Col-0 plants, including the CG, CNG, and CHH sites, while the decrease in DNA methylation in the *GSTF14* promoter in cold stress-treated *ros1* mutants was partially inhibited (Fig. 4C). When Col-0 plants were used as the control, the DNA methylation levels of the repeats in the *GSTF14* promotor in *ros1* mutants were not significantly altered (Fig. 4C). These results revealed that activation of the expression of the defense gene *ACD6* in the SA pathway, the stress response genes *GSTF14* and *ACO3* was related to ROS1-mediated DNA demethylation.

## Discussion

Scientists began to focus on the important role of hormones in the regulation of plant growth and development and resistance to abiotic stresses in 1930. In this field, the ABA pathway has been well studied. ABA is a key hormone regulating the response of plants to abiotic stresses, such as drought. A total of 40 stress-inducible transcription factor genes have been found in *Arabidopsis* [35]. For example, MYB transcription factors are indispensable to the adaptation of plants to cold stress and can affect plant resistance to drought by controlling stress-induced ABA synthesis [36]. We know less about the role of the SA defense pathway in the response of plants to abiotic stresses and the related molecular mechanisms.

In this study, we demonstrated that abiotic stresses, including water deficit, cold, and salt stresses, induced DNA demethylation of repeats in the promoters of *ACD6*, *ACO3*, and *GSTF14* and transcriptionally activated their expression. Furthermore, our results confirmed that ROS1-mediated DNA demethylation plays a role in the process of transcriptional activation of the target genes (*ACD6*, *ACO3*, and *GSTF14*) regulated by RNA-directed DNA methylation (RdDM) when *Arabidopsis* Col-0 plants are subjected to cold stress.

Sequencing results confirmed that the increase in the expression of *ACD6*, *GSTF14*, and *ACO3* was related to the reduction in the DNA methylation levels of the promoters of these genes. Under the same stress conditions, different genes differ in the levels and patterns of DNA methylation (Fig. 2), suggesting that complex molecular mechanisms regulate the expression of these genes. Our results revealed that abiotic stresses (water deficit, cold, and salt stresses) induced DNA demethylation of the *ACD6*, *ACO3* and *GSTF14* promoters and transcriptionally activated the expression of the defense genes *ACD6*, *NPR1* and *PR5* in the SA pathway and stress response genes *ACO3* and *GSTF14*, thereby enhancing the adaptability of plants to abiotic stresses.

ROS1 can negatively regulate the RdDM pathway [8, 9]. Recent research has shown that ROS1-mediated DNA demethylation can act on three DNA methylation sites: CG, CNG, and CHH [37]. DNA methylation sequencing of *ros1* mutants has revealed that ROS1 generally targets genes containing CG, CNG, and CHH methylation sites in transposable elements and repeats but does not target genes containing only CG methylation sites [38]. Furthermore, our results confirmed that the RdDM pathway has an important role in maintaining the low transcription levels of *ACD6*, *GSTF14*, and *ACO3* in wild-type Col-0 plants (Fig. 3A, B). When cold stress-treated Col-0 plants were used as the control, the increase in the expression of *ACD6*, *GSTF14*, and *ACO3* in *ros1* mutants treated with cold stress for 24 h was partially inhibited (Fig. 3D). Furthermore, after 24 h of cold stress treatment in Col-0 plants, DNA methylation levels in the repeats of the *ACD6*, *ACO3* and *GSTF14* promoters were significantly reduced, while the decrease in DNA methylation levels in the repeats of the *ACD6* and *ACO3* promoters in cold stress-treated *ros1* mutants was partially inhibited (Fig. 4). These data analyses indicate that ROS1 is only partially responsible for changes in expression of and levels of methylation of the target genes under cold stress.

This study has revealed the role of ROS1 in the regulation of defense genes *ACD6*, *NPR1* and *PR5* in the SA pathway and *ACO3* and *GSTF14* in response to abiotic stresses. Due to the complexity of the dynamic regulation of DNA methylation, the molecular mechanisms by which plants adapt to various adverse environmental factors and how different signaling pathways interact still require in-depth study.

## Conclusions

Our study reveals the molecular mechanism by which plant defense genes in the SA pathway and stress resistance genes are involved in responses to various abiotic stresses. The results show that the RdDM pathway has an important role in maintaining the low transcription levels of *ACD6*, *GSTF14*, and *ACO3* in wild-type Col-0 plants. Further studies revealed that abiotic stresses induced DNA demethylation of the *ACD6*, *ACO3*, and *GSTF14* promoters and transcriptionally activated the expression of defense genes and stress resistance genes. Moreover, ROS1-mediated DNA demethylation plays an important role in this process.

## Methods

### Aabiotic stress treatments and hydrogen peroxide content measurements

*thaliana* ecotype Columbia (Col-0) and mutant plants were used for this work. The *ago4* mutant seeds (original source) [39] and *ros1* and *rdd* mutant seeds (original source) [40] were provided by Chengguo Duan in Shanghai Center for Plant Stress Biology, Shanghai Institute of Biological Sciences, Chinese Academy of Sciences (CAS). Col-0, *met1*, *drm1/2*, and *cmt3* mutant seeds were provided by the Institute of Genetics and Developmental Biology, CAS. Seeds were surface-sterilized with 30% bleach, washed three times with sterile water, and sown on Murashige and Skoog (MS) plates. The seedlings were grown for approximately 2 weeks and then transferred to a 22 °C environment with a 16-h light/8-h dark cycle for 2 weeks. *Arabidopsis* plants were transferred to soil in a greenhouse (22 °C, with a 16-h light/8-h dark cycle) and treated with abiotic stresses, including cold stress (4 °C, 24 h), salt stress (150 mM NaCl, 3 days), and water deficit stress (not watered, 7 days). The measurements of hydrogen peroxide content were performed by spectrophotometry [41] after Col-0 plants were treated with cold stress, water deficit or salt stress, respectively [34]. Each experiment consisted of three biological replicates and was repeated twice. The significant experimental details are as follows.

**Table:** 

Cold stress	Salt stress	Water deficit stress
4 °C	150 mM NaCl	not watered
24 h	3 days	7 days

### RT-qPCR analysis and RNA gel blot analysis

Total RNA was isolated using TRIzol reagent (Invitrogen) according to the manufacturer’s protocols. Total RNA was subsequently used for RT-qPCR analysis. For RT-qPCR, total RNA was extracted from the treated plants and subsequently used for reverse transcription. Complementary DNA synthesis was performed using a reverse transcription kit (Takara). Quantitative RT-PCR was performed using SYBR green mix (Qiagen). Each experiment consisted of three biological replicates and was repeated twice. For the high-molecular-weight RNA gel blot analyses, 10 mg of total RNA was extracted from the treated plants and separated on 1% agarose-formaldehyde gels, transferred to Hybond-Nþ membranes, and hybridized as described previously [4]. ACD6 (AT4G14400) and GSTF14 (AT1G49860) probe primer pairs were as follows: F (ACD6), 5′-TCTCCCTGGTGAAGATGTCG-3′ and R (ACD6), 5′-TTACCGATGCAACAAGAGCC-3′; F (GSTF14), 5′-AGGCGAGTCTCCTTACT TGG-3′ and R (GSTF14), 5′-TTATAGGCAAACGACGCTGC-3′; F (ACO3), 5′-ACGAGTCAA TCACCAAGGGT-3′ and R (ACO3), 5′-GAAGTCCTTACGGT CAACGC-3′.

### Bisulfite sequencing

Total DNA was extracted using cetyl trimethyl ammonium bromide (CTAB) buffer as previously described [23] and purified using a DNA purification kit (Promega). The purified DNA was used for bisulfite treatment using the EpiTect bisulfite kit (Qiagen, http://www.qiagen.com/default.aspx) according to the manufacturer’s instructions. The purified bisulfite-treated DNA was amplified by *ACD6* (AT4G14400) and *GSTF14* (AT1G49860) promoter-specific primer pairs as follows:

F (*ACD6*), 5′-AAGTTTATTGATGAAAGGAG-3′ and R (ACD6), 5′-CTTACTT (G/A) TCTT CATCAA-3′; F (GSTF14), 5′-TTTGAAAGTTGGTGTATTAAA-3′ and R (GSTF14), 5′-CCCATA CCTATCATATTTCAT-3′; F (ACO3), 5′-GTAATATTAGTAAAGATGTGT-3′ and R (ACO3), 5′-CACTACTTTC ATTATACTCTTT-3′. PCR included 40 cycles of 95 °C for 30 s, 55 °C for 30 s, 50 °C for 30 s, and 62 °C for 2 min. Cytosine methylation analysis was provided by https://www.cymate.org/cymate.html, as described previously [42]. Each experiment consisted of three biological replicates and was repeated twice.

## Supplementary Information

**Additional file 1.**

**Additional file 2.**

**Additional file 3.**

## Data Availability

The datasets supporting the conclusions of this article are included within the article and its additional files. About genes database could download from NCBI by their accession number. The accession numbers of these genes are as follows: *ACD6 (AT4G14400)* and *GSTF14 (AT1G49860)*.

## Abbreviations

SA: Salicylic acid
ABA: Abscisic acid
JA: Jasmonic acid
ET: Ethylene
ACD6: Accelerated cell death 6
ACO3: Aconitate hydratase 3
GST: Glutathione S-transferase
DCL3: Dicer-like 3
RdDM: RNA-directed DNA methylation
siRNAs: Small interfering RNAs
AGO4: Argonaute protein 4
ROS1: Repressor of silencing 1
Pol IV: RNA polymerase IV
RDR2: RNA-dependent RNA polymerase
RT-qPCR: Reverse transcription-quantitative PCR
Col-0: Columbia
RT-sqPCR: Reverse transcription-semiquantitative PCR
*rdd*: *Ros1 dml2 dml3*
