# Supplementary figures and images for: ROS1-mediated decrease in DNA methylation and increase in expression of defense genes and stress response genes in Arabidopsis thaliana due to abiotic stresses
Source: BMC Plant Biol. 2022 Mar 7;22:104. doi: 10.1186/s12870-022-03473-4 (PMC8903643; doi:10.1186/s12870-022-03473-4)

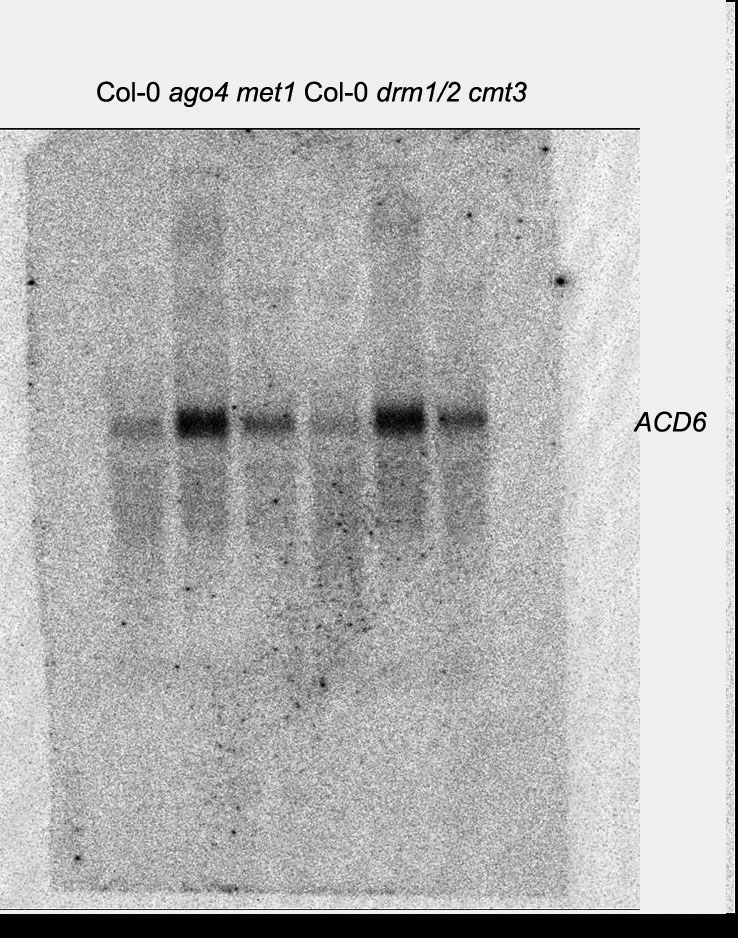

Supplement: Supplementary file 1 — Additional file 1. [file 12870_2022_3473_MOESM1_ESM.jpg]

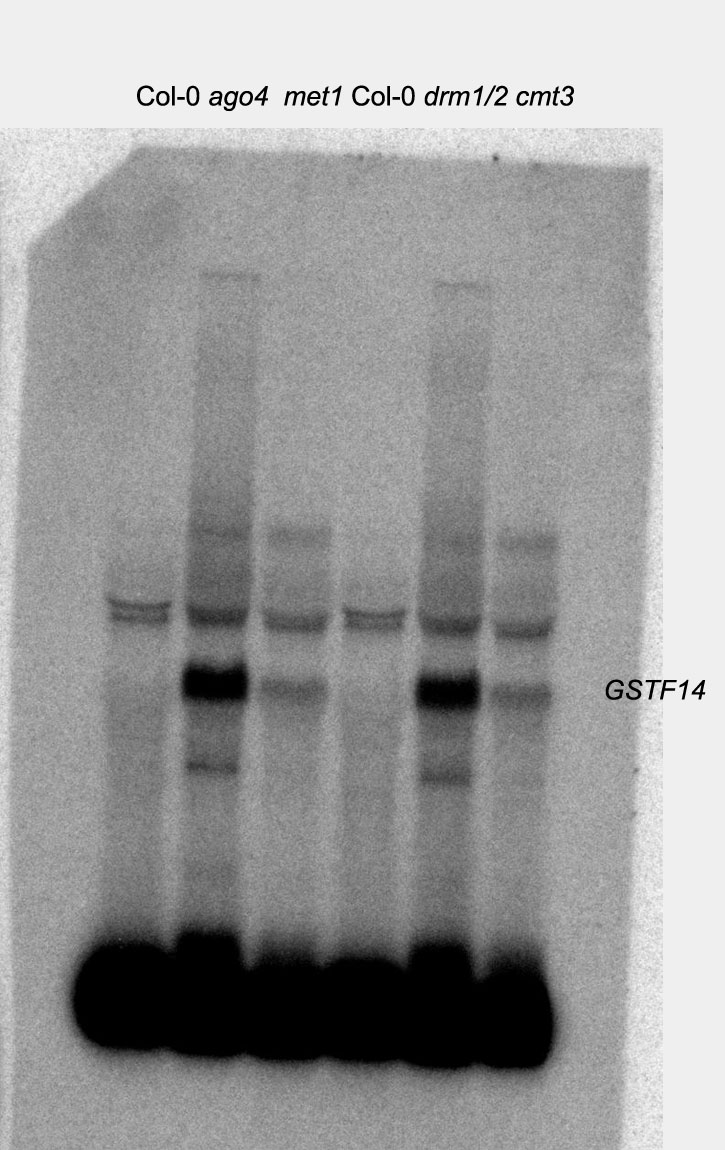

Supplement: Supplementary file 2 — Additional file 2. [file 12870_2022_3473_MOESM2_ESM.jpg]

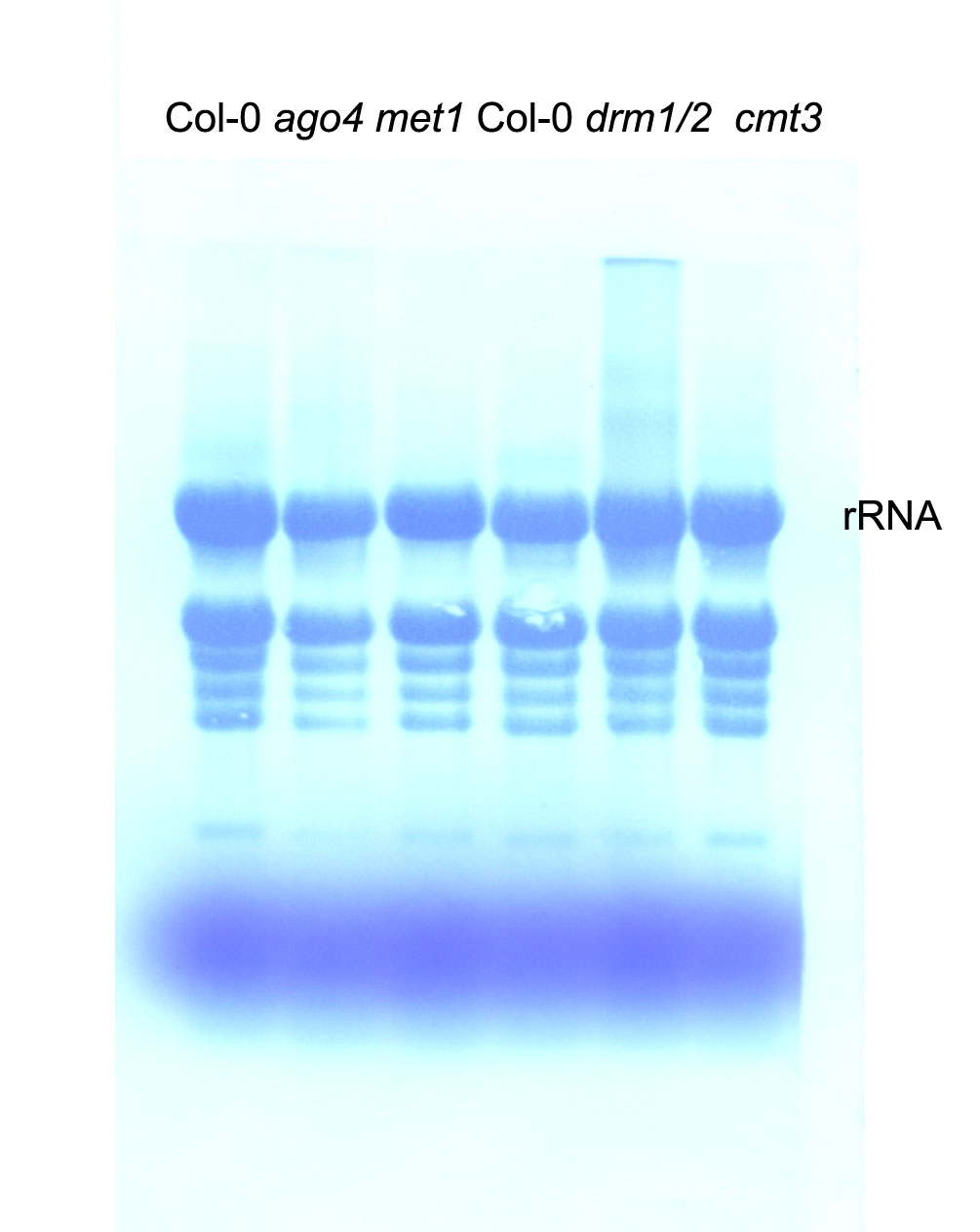

Supplement: Supplementary file 3 — Additional file 3. [file 12870_2022_3473_MOESM3_ESM.jpg]
